# No Association between Loss-of-Function Mutations in *filaggrin* and Diabetes, Cardiovascular Disease, and All-Cause Mortality

**DOI:** 10.1371/journal.pone.0084293

**Published:** 2013-12-18

**Authors:** Lise Lotte N. Husemoen, Tea Skaaby, Torben Jørgensen, Jacob P. Thyssen, Michael Meldgaard, Pal B. Szecsi, Steen Stender, Jeanne Duus Johansen, Allan Linneberg

**Affiliations:** 1 Research Centre for Prevention and Health, Copenhagen University Hospital Glostrup, Glostrup, Denmark; 2 Faculty of Health Science, University of Copenhagen, Copenhagen, Denmark; 3 National Allergy Research Centre, Department of Dermato Allergology, Copenhagen University Hospital Gentofte, Hellerup, Denmark; 4 Department of Clinical Biochemistry, Copenhagen University Hospital Gentofte, Hellerup, Denmark; University of Pennsylvania, United States of America

## Abstract

**Background:**

Common loss-of-function mutations in the filaggrin gene (FLG) are a major predisposing risk factor for atopic disease due to reduced epidermal filaggrin protein levels. We previously observed an association between these mutations and type 2 diabetes and hypothesized that an inherited impairment of skin barrier functions could facilitate low-grade inflammation and hence increase the risk of diabetes and cardiovascular disease. We examined the association between loss-of-function mutations in *FLG* and diabetes, stroke, ischemic heart disease (IHD), and all-cause mortality in the general population.

**Methods:**

The R501X and 2282del4 loss-of function mutations in *FLG* were genotyped in four Danish study populations including a total of 13373 adults aged 15-77 years. Two of the studies also genotyped the R2447X mutation. By linkage to Danish national central registers we obtained information for all participants on dates of diagnoses of diabetes, stroke, and IHD, as well as all-cause mortality. Data were analyzed by Cox proportional hazard models and combined by fixed effect meta-analyses.

**Results:**

In meta-analyses combining the results from the four individual studies, carriage of loss-of-function mutations in *FLG* was not associated with incident diabetes (hazard ratio (HR) (95% confidence intervals (CI)) = 0.95 (0.73, 1.23), stroke (HR (95% CI) = 1.27 (0.97, 1.65), ischemic heart disease (HR (95%CI) = 0.92 (0.71, 1.19), and all-cause mortality (HR (95%CI) = 1.02 (0.83, 1.25)). Similar results were obtained when including prevalent cases in logistic regression models.

**Conclusion:**

Our results suggest that loss-of-function mutations in *FLG* are not associated with type 2 diabetes, cardiovascular disease, and all-cause mortality. However, larger studies with longer follow-up are needed to exclude any associations.

## Introduction

Filaggrin is a major structural protein in the upper epidermis [1]. Several loss-of-function mutations have been identified in the filaggrin gene (*FLG*), which result in filaggrin deficiency and impairment of the skin barrier. These gene defects are quite common and found in 7-10% of the white European general population [2–4]. The 2282del4 and R501X are the most frequent and comprise 80% of known loss-of-function genetic mutations in *FLG* [5]. They are believed to have equivalent biological effect, and are strong predisposing genetic risk factors for atopic dermatitis and also associated with other allergic diseases such as food allergy, asthma and rhinitis [5–7]. Whether these common loss-of-function mutations have an effect on other chronic diseases is currently unknown. Previous studies have suggested a possible association with type 2 diabetes and CVD [2,3,8]. On one hand, it has been hypothesized that impairment of the skin barrier increases the propensity to develop low-grade inflammation, which could increase the risk of diabetes and CVD [2]. On the other hand, it has also been suggested that loss-of function mutations in *FLG* protects against diabetes and CVD by increasing blood vitamin D concentrations. Hence, loss-of function mutations in *FLG* have been shown to result in higher serum vitamin D concentrations [9] supposedly due to a decreased UV protection of the keratinocytes [10]. Low serum 25(OH)D has been associated with risk of diabetes [11] and CVD in observational studies.

Here, we examined whether loss-of-function mutations in *FLG* were associated with diabetes, IHD, stroke, and all-cause mortality by combining data from four Danish studies.

## Materials and Methods

### Study population

We used data from four Danish studies conducted at the Research Centre for Prevention and Health in the Copenhagen area: the Health2006 study [12], the Inter99 study [13], the Copenhagen Allergy 1998 study [14], and the Monica10 study [15]. All participants were identified in the central Danish Civil Registration System, and then recruited by invitation.

The Health2006 took place during 2006-2008 and consisted of a random sample of 7931 men and women aged 18-69 years (Danish nationality and born in Denmark) invited to participate in a general health examination with 3471 (43.8%) participating. The aim was to investigate the prevalence and risk factors of chronic diseases such as mental health, asthma, allergies, CVD, and diabetes.

The Inter99 study is a randomised controlled trial (CT00289237, ClinicalTrials.gov) investigating the effects of lifestyle intervention on CVD (N=61301)[13]. We used baseline data from a random subsample of 12934 men and women aged approximately 30, 35, 40, 45, 50, 55, or 60 years invited to participate in a health examination during 1999-2001. A total of 6784 (52.5%) participated. Only participants originating from Denmark, Norway, Sweden, Iceland, and the Faeroe Islands were included (n=6405) [16]. 

The Copenhagen Allergy study was initiated in 1990 and includes a group of participants randomly selected from the general population and a selected group of participants with allergic respiratory symptoms (recruited from a random sample of the general population by a screening questionnaire). We used data from the re-examination in 1997-1998 where a total of 1966 men and women aged 15–77 years with Danish nationality were invited for a health examination. A total of 1216 (61.9%) participated.

The Danish Monitoring Trends and Determinants of Cardiovascular Disease (MONICA)10 cohort consists of 4130 individuals with Danish >Nationality, who were invited to participate in a follow-up examination during 1993-1994. A total of 2656 (64%) aged approximately 41, 51, 61, or 71 years old participated .

All participants underwent a general health examination including objective assessments and tests, self-administered questionnaires, blood samples, and DNA. Participant without valid FLG genotype were excluded leaving 3343 (96.3%) (Health2006), 6247 (97.5%) (Inter99), 1206 (99.2%) (Allergy1998), and 2577 (97.0%) (MONICA10) participants for statistical analysis (n_total_= 13373).

### Ethics statement

Informed written consent was obtained from all participants. The studies have been approved by the Ethical Committee of Copenhagen, and have been performed in accordance with the 1964 Declaration of Helsinki and its later amendments.

### Genotyping of FLG

In the health2006 and Inter99 cohorts, we determined the three most common loss-of-function mutations in *FLG* (R501X, 2282del4, and R2447X), where the first two have been shown to be associated with higher serum 25(OH)D concentrations[9]. Regions covering these mutations were amplified from genomic DNA by allele-specific, asymmetric PCR with tagged and biotinylated primers. The obtained PCR products were hybridized to spectrally coded microbeads (Luminex, Austin, Texas) carrying tag-sequences as DNA probes, and subsequently analyzed on a BioPlex 200 (Bio-Rad Laboratories, Hercules, CA). The call rates were >99% for all six alleles [17].

Only the two most common loss-of-function mutations in *FLG* (R501X, 2282del4) were determined in the Allergy1998 and MONICA10 cohorts using the same methods.

Due to low numbers of the specific loss-of-function mutations in *FLG* and few compound heterozygotes, participants, who were either heterozygous or homozygous for at least one of the determined loss-of-function mutations in *FLG*, were categorized as loss-of-function mutation in *FLG* carriers.

### Diabetes

Information on diabetes was obtained from the Danish National Diabetes Register. This registry uses five diagnostic criteria: hospitalization with a diagnosis of diabetes (International Classification of Disease (ICD)-8: 249 or 250, ICD-10: DE10-14, DH 36.0 or DO24 (excluding D=24.4)); registration of chiropody (coded for diabetes) in the National Health Insurance Service Registry; frequent measurements of blood glucose either at least five times within 365 days (1 year), or at least two annual measurements of glucose during a 5-year period (registered in the National Health Insurance Service Registry); and prescription of insulin or oral anti-diabetic medication at least twice (registered in the Register of Medicinal Product Statistics) [18]. If one of these criteria is met the subject is classified as having diabetes.

A baseline history of diabetes was defined as a record in the diabetes register prior to the date of the baseline examination, or as self-report of diabetes at baseline. Incident diabetes was defined as new cases of diabetes during follow-up among those without history of diabetes. The registry does not distinguish between subtypes of diabetes, but given the age of the participants most incident cases are believed to be type 2 diabetes. Participants were followed from the date of the health examination to 31 December 2011, or to the date at which the individual emigrated, died, or developed diabetes, whichever date came first. Information on emigrations and deaths was obtained from the Danish Civil Registration System.

### Stroke and IHD

Information on stroke and IHD was obtained by linkage to the Danish National Hospital Register [19] and the Danish Registers of Causes of Death [20] using the participants unique personal identification number (CPR numbers), which allows linkage on an individual level of data from complete national registers. The Danish National Hospital Register has information on all admissions to Danish hospitals since 1977. Each admission is registered by one primary diagnosis and optionally one or more secondary diagnoses according to the International Classification of Diseases (ICD). Likewise, The Danish Registers of Causes of Death provide up to three diagnoses suspected to be the cause of death. Since Denmark never used the ICD9 but went directly from the ICD8 to the ICD10, the included diagnoses defining a stroke were ICD10: I60–I69 and ICD8: 430-431, 433–434, and 436, and the included diagnoses defining IHD were: ICD10: I20-I25 and ICD8: 410-414.

A baseline history of stroke and IHD, respectively, was defined as a record of stroke or IHD in the central registers prior to the date of the baseline examination, or self-reported history of physician-diagnosed myocardial infarction or ischemic or haemorrhagic stroke at baseline. Incident stroke or IHD was defined as new cases during follow-up among those without a baseline history of stroke or IHD, respectively.

Participants without a history of stroke or IHD were followed from the date of the health examination until 31 December 2011, or to the date at which the individual had a stroke or IHD, died from other causes, or emigrated whichever date came first. Information on emigrations and deaths from other causes was obtained from the Danish Civil Registration System. A few cases (i.e. individuals who died from stroke or IHD between 31 December 2010 and 31 December 2011 without prior hospitalisation and thereby registration in Danish National Hospital Register) may have been miss-classified, since information on causes of death was only available until 31 December 2010.

### All-cause mortality

Information on all-cause mortality was obtained from the Danish Civil Registration System. Participants were followed from the date of the health examination until 17 December 2012, or to the date at which the individual died, or emigrated, whichever date came first.

### General characteristics

Body mass index (BMI) was calculated as weight divided by height squared. The self-administered questionnaires provided information on lifestyle habits, medical history and socio-demographic variables. Smoking status was categorised as current smokers (daily and occasional smokers) and non-smokers (never and ex-smokers). Type and weekly average amount of alcoholic beverages were recorded and average number of standard (approx. 1.5 cl or 12 g ethanol) drinks per week calculated. Physical activity during leisure time was reported as sedentary, light, moderate, or vigorous. Finally, participants were asked about history of doctor diagnosed chronic diseases.

### Statistical analyses

Statistical analyses were performed using SAS, version 9.3 (SAS Institute Inc, Cary, NC, USA) and stata, version 12.1 (StataCorp LP, College Station, Texas, USA). All reported P values are two-tailed and statistical significance was defined as p<0.05. Cox regression analyses were used to examine the association with incident disease (diabetes, stroke, or IHD) and all-cause mortality. Individuals with history of disease (diabetes, stroke, or IHD) were excluded. Estimates are presented as hazard ratios (HR) with 95% confidence intervals (95% CI). We used delayed entry and age as the underlying time axis. The few participants lost to follow-up (i.e. those who emigrated or died) contributed to the risk time until the date of their last registered activity. Cox models were adjusted for sex. Individuals with a baseline history of disease were included as cases in supplemental analyses using logistic regression adjusted for age and sex. Results from the four individual studies (Health2006, Inter99, Allergy1998, and MONICA10) were pooled by the inverse variance method in fixed effects meta-analyses. Random effects meta-analyses were also performed to confirm the results. The I^2^ test was used to examine heterogeneity between studies.

## Results

The prevalence of loss-of-function mutations in *FLG* ranged from 7.1% in the Allergy1998 study to 8.9% in the Health2006 study, and an average of 8.4%. Minimum-maximum incidences of diabetes, IHD, stroke, and all-cause mortality across the four studies were 3.5-7.4, 4.7-9.0, 2.9-7.6, and 2.8-18.8 per 1000 person-years at risk, respectively. Further characteristics of the study participants are given in table 1. The frequency of individual loss-of-function mutations in *FLG* are given in table 2. None of the examined mutations in *FLG* deviated from the Hardy-Weinberg equilibrium (table 2).

**Table 1:** Characteristics of the Health2006, Inter99, Allergy1998, and MONICA10 study populations.

	**Health2006**	**Inter99**	**Allergy1998**	**MONICA10**
**Baseline examination year**	2006-2008	1999-2001	1997-1998	1993-1994
**Number of individuals**	3343	6247	1206	2577
**Loss-of function mutations in *FLG*, % (n/nt_otal_)**	8.9 (296/3343)	8.8 (551/6247)	7.1 (86/1206)	7.5 (194/2577)
**Male gender, % (n/n_total_)**	44.7 (1495/3343)	48.7 (3039/6247)	45.7 (551/1206)	49.9 (1286/2577)
**Age in years, mean (SD)**	49.39 (13.03)	46.23 (7.91)	40.08 (15.10)	55.30 (10.75)
**Daily smokers, % (n/n_total_)**	22.7 (750/3311)	35.6 (2214/6215)	33.3 (401/1204)	42.0 (1079/2577)
**BMI in kg/m^2^, mean (SD)**	25.93 (4.71)	26.28 (4.62)	26.65 (4.58)	25.94 (4.20)
**Alcohol in drinks/week, mean (SD)**	9.69 (10.20)	10.45 (13.30)	6.79 (8.04)	9.99 (12.20)
**Low physical activity, % (n/n_total_)**	18.5 (611/3308)	20.7 (1273/6137)	26.1 (313/1200)	21.2 (536/2534)
**Asthma, % (n/n_total_)**	10.8 (354/3288)	8.6 (518/6033)	6.5 (78/1204)	7.4 (190/2572)
**Hay fever, % (n/n_total_)**	18.0 (590/3281)	Not available	11.2 (135/1205)	11.2 (289/2574)
**Atopic dermatitis, % (n/n_total_)**	5.4 (176/3248)	Not available	5.3 (64/1205)	2.5 (63/2571)
**History of diabetes, % (n/n_total_)**	5.0 (168/3343)	2.2 (139/6247)	2.2 (26/1206)	2.9 (75/2577)
**History of IHD, % (n/n_total_)**	3.1 (105/3343)	1.6 (102/6247)	2.3 (28/1206)	6.0 (154/2577)
**History of stroke, % (n/n_total_)**	3.3 (82/3343)	1.3 (83/6247)	1.3 (16/1206)	5.0 (129/2577)
**Incidence diabetes, per 1000 person-years (n/n_person-years_)**	3.5 (49/14157)	5.4 (380/69918)	4.3 (65/15282)	7.4 (276/37369)
**Incidence IHD, per 1000 person-years (n/n_person-years_)**	4.9 (71/14402)	4.8 (340/70901)	4.7 (71/15199)	9.0 (328/36537)
**Incidence stroke, per 1000 person-years (n/n_person-years_)**	3.4 (50/14562)	2.9 (207/71839)	3.8 (59/15495)	7.6 (283/37359)
**All-cause mortality, per 1000 person-years (n/n_person-years_)**	2.8 (51/18189)	3.7 (291/79370)	6.1 (103/16952)	18.8 (787/41883)

**Table 2:** Prevalence of *FLG* loss-of-function mutations genotyped in the Health2006, Inter99, Allergy1998, and MONICA10 study populations.

**Study**	**Loss of function mutations in *FLG***	***FLG* genotypes, n**			**P value** ^a^
		**wildtype**	**heterozygotes**	**minor homozygotes**	
**Health2006**	**R501X**	3236	110	3	0.076
	**2282del4**	3190	155	4	0.13
	**R2447x**	3312	30	1	0.072
**Inter99**	**R501X**	6034	214	1	1.00
	**2282del4**	5971	277	0	0.077
	**R2447x**	6184	64	0	1.00
**Allergy1998**	**R501X**	1165	41	0	1.00
	**2282del4**	1160	45	1	0.37
**MONICA10**	**R501X**	2504	71	1	0.40
	**2282del4**	2455	123	0	0.40

^a^ Exact P value from Hardy Weinberg equilibrium test.

**Figure 1:**
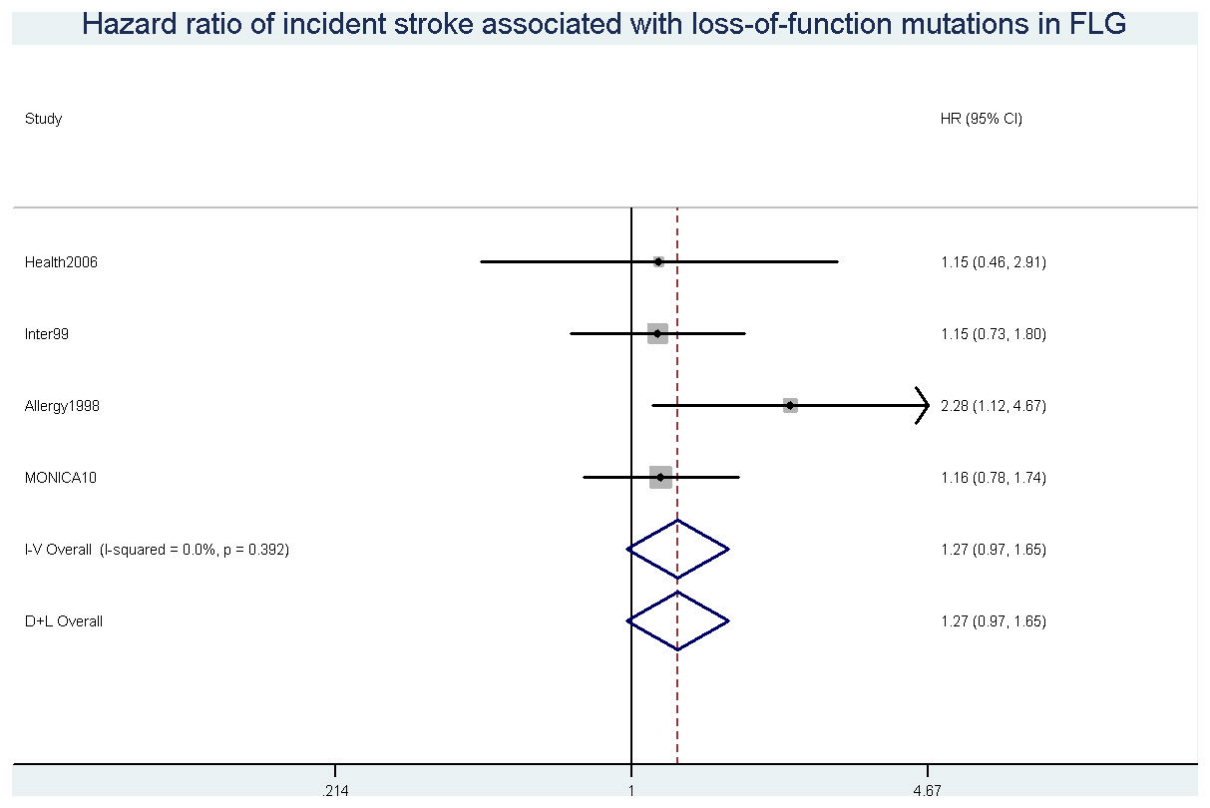
Hazard ratio of incident stroke associated with loss-of-function mutations in *FLG*.

A non-significant association was observed between loss-of function mutations in *FLG* carriage and risk of incident stroke (HR (95% CI) = 1.27 (0.97, 1.65), p=0.079) (Figure 1). 

No statistically significant associations were observed between loss-of function mutations in *FLG* and incidence of type 2 diabetes (HR (95% CI) = 0.95 (0.73, 1.23), p= 0.68) (Figure S1), IHD (HR (95% CI) = 0.92 (0.71, 1.19), p=0.52) (Figure S2), and all-cause mortality (HR (95% CI) = 1.02 (0.83, 1.25), p=0.87) (Figure S3), respectively. I^2^ ranged from 0.0% to 9.4 % in meta-analyses with incident outcomes indicating low heterogeneity between studies (range p-values: 0.35-0.85). Accordingly similar results were obtained with random effects meta-analyses.

**Figure 2:**
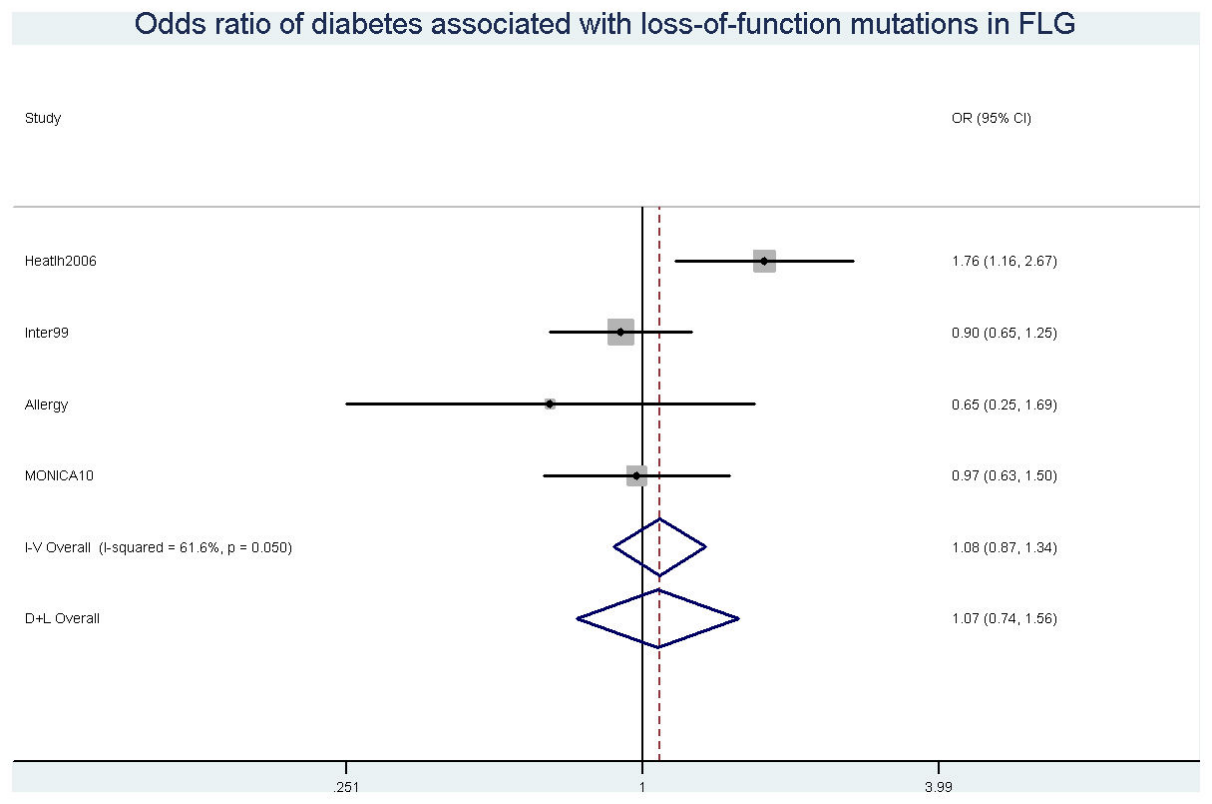
Odds ratio of diabetes associated with loss-of-function mutations in *FLG*.

When including historical cases, loss-of-function mutation in *FLG* carriage was associated with an increased risk of diabetes in the Health2006 study (OR (95% CI) = 1.76 (1.16, 2.67), p=0.011) (Figure 2). However, no association was observed in the overall meta-analysis (OR (95% CI) = 1.08 (0.87, 1.34), p=0.47) (Figure 2). Also no statistically significant associations were observed when including historical cases of stoke (overall OR (95% CI) = 1.19 (0.93, 1.52), p=0.17) (Figure S4) and IHD (overall OR (95% CI) = 0.89 (0.71, 1.13), p=0.34) (Figure S5). When doing separate analyses by mutation, a statistical significant association was observed between incident stroke and the 2282del4 loss-of-function mutation in *FLG* (overall HR (95% CI) = 1.42 (1.03, 2.00), p=0.032), but not with the R501X mutation (overall HR (95% CI) = 1.12 (0.74, 1.72), p=0.83). Neither R501X nor 2282del4 were associated with diabetes, IHD, or all-cause mortality (Table S1).

## Discussion

We found no consistent associations between loss-of-function mutations in *FLG* and either incident cases of diabetes, IHD, stroke or all-cause mortality when examining four Danish studies including a total of 13,373 adults retrieved from the general population. This suggests that carriage of loss of function mutations in *FLG* resulting in a dysfunctional skin barrier does not affect cardiovascular risk as previously proposed [2,3]. Neither does it seem to increase all-cause mortality.

We have previously reported on a possible association between loss-of-function mutations in *FLG* and self-reported diabetes in a cross-sectional study based on the Health2006 cohort [2]. In the current study, we confirmed this association for the Health2006 cohort using an objective registry-based definition of diabetes. However, the association could not be confirmed when restricting the analysis to incident cases of diabetes, or when expanding the study population to include the inter99, Allergy, MONICA10 studies. This suggests that the previously reported association in the Health2006 cohort may have been a chance finding.

Moreover our results are not supportive of previously reported associations between loss-of-function mutations in *FLG* and a more favourable lipid profile [3] as well as lower blood adiponectin concentrations [8]. These associations were assumed to be mediated via vitamin D, and loss-of function mutations in *FLG* resulting in higher vitamin D hypothesised to result in lower diabetes and cardiovascular risk, which was not observed in the current study. In agreement with the current study, no associations with the intermediate risk factors blood pressure and obesity were observed [3].

Nonetheless, previous studies have found an inverse association between type 1 diabetes and atopic dermatitis [21,22] also suggestive of a protective role of loss-of-function mutations in *FLG* in diabetes. Given the age of the participants in the current study most incident cases are likely of type 2, and our results are not in contrast with a potential inverse association between mutations in *FLG* and type 1 diabetes.

Interestingly, our results suggests that carriage of loss-of-function mutations in *FLG* does not increase all-cause mortality, despite the well known causal association with atopic dermatitis and atopic diseases such as hay fever and asthma [1]. However, atopic diseases are associated with significant morbidity for patients and their families, but generally are not lethal as such. Also, loss of function mutations in *FLG* have been associated with increased blood concentrations of vitamin D which may counterbalance any detrimental effects [9].

Moreover, in supplemental analyses by mutation, we did observe a statistical significant association between one of the loss-of-function mutations in FLG and stroke. This may suggest that all the mutations do not act the same, but it could also be a chance finding, or due to another causal variant in high linkage disequilibrium.

The strengths of our study are the objectively measured outcomes and the relatively long follow-up, which allow us to examine incident cases. Also linkage to Danish national registers enabled almost complete follow-up, and very few missing data. The registers have very high validity, although participants with undiagnosed diabetes would have been misclassified.

Also our study was based on a relatively large sample size obtained by combining four study populations recruited from the same geographical region of Denmark. However despite more than 13.000 participants, the relatively wide confidence intervals indicate, that false negative findings due to low power, cannot be fully excluded. A further possible explanation for the lack of association is that the data include many relatively young individuals who would not be expected to have developed these diseases or died yet. Moreover, the data represent samples from selected studies each of which could introduce bias. 

Other limitations of the study are the risk of bias that may arise if *FLG* genotypes were unequally distributed among the persons that died before the study. However, since the examined loss-of-function mutations in *FLG* did not deviate significantly from the hardy Weinberg equilibrium, this potential bias is likely to be minimal. Furthermore bias may have occurred if non-participant differed from participant in a differential manner. 

Moreover, only two mutations in *FLG* were available for the MONICA10 and the Allergy cohorts, which may have lead to some misclassification, and attenuation toward the null value. However, the R2447X mutation is uncommon compared to the two other mutations available on all cohorts, suggesting that the effect is small.

Finally, most FLG mutation carriers were heterozygotes individuals. It is possible that larger effects would have been observed, if more compound heterozygote and homozygote individuals were available.

In conclusion, our results suggest that loss-of-function mutations in *FLG* are not associated with type 2 diabetes, cardiovascular disease, and all-cause mortality. However, further studies with longer follow-up and larger numbers are needed to exclude any associations.

## Supporting Information

Table S1 **Association of R501X and 2282del4 loss-of-function mutations in *FLG* and diabetes, stroke, IHD, and all-cause mortality.**
(DOC)

Figure S1 **Hazard ratio of incident diabetes associated with loss-of-function mutations in *FLG*.**
(TIF)

Figure S2 **Hazard ratio of incident IHD associated with loss-of-function mutations in *FLG*.**
(TIF)

Figure S3 **Hazard ratio of all-cause mortality associated with loss-of-function mutations in *FLG*.**
(TIF)

Figure S4 **Odds ratio of stroke associated with loss-of-function mutations in *FLG*.**
(TIF)

Figure S5 **Odds ratio of IHD associated with loss-of-function mutations in *FLG*.**
(TIF)
