# Supplementary material for: No Association between Loss-of-Function Mutations in filaggrin and Diabetes, Cardiovascular Disease, and All-Cause Mortality
Source: PLoS One. 2013 Dec 18;8(12):e84293. doi: 10.1371/journal.pone.0084293 (PMC3867483; doi:10.1371/journal.pone.0084293)
Supplement: Table S1 — Association of R501X and 2282del4 loss-of-function mutations in FLG and diabetes, stroke, IHD, and all-cause mortality. (DOC) [file pone.0084293.s001.doc]

Table S1. Association of R501X and 2282del4 loss-of-function mutations in *FLG* and diabetes, stroke, IHD, and all-cause mortality.

| **Outcome** | **Loss-of-function mutations in *FLG*** | **HR (95% CI), P value** | **OR (95% CI) , P value** |
| --- | --- | --- | --- |
| **Diabetes** | **R501X** | 1.03 (0.69, 1.55), 0.88 | 1.11 (0.80, 1.54), 0.53 |
|  | **2282del4** | 1.07 (0.77, 1.50), 0.68 | 1.15 (0.86, 1.54), 0.36 |
|  |  |  |  |
| **Stroke** | **R501X** | 1.12 (0.72, 1.74), 0.83 | 1.09 (0.73, 1.61), 0.68 |
|  | **2282del4** | 1.43 (1.03, 2.00), 0.032 | 1.34 (0.98, 1.84), 0.066 |
|  |  |  |  |
| **IHD** | **R501X** | 0.99 (0.67, 1.48), 0.97 | 1.14 (0.81, 1.61), 0.44 |
|  | **2282del4** | 0.89 (0.63, 1.26), 0.50 | 0.73 (0.52, 1.02), 0.066 |
|  |  |  |  |
| **Mortality** | **R501X** | 1.04 (0.75, 1.45), 0.81 | not available |
|  | **2282del4** | 0.99 (0.76, 1.29), 0.96 | nor available |
