# Supplementary figures and images for: No Association between Loss-of-Function Mutations in filaggrin and Diabetes, Cardiovascular Disease, and All-Cause Mortality
Source: PLoS One. 2013 Dec 18;8(12):e84293. doi: 10.1371/journal.pone.0084293 (PMC3867483; doi:10.1371/journal.pone.0084293)

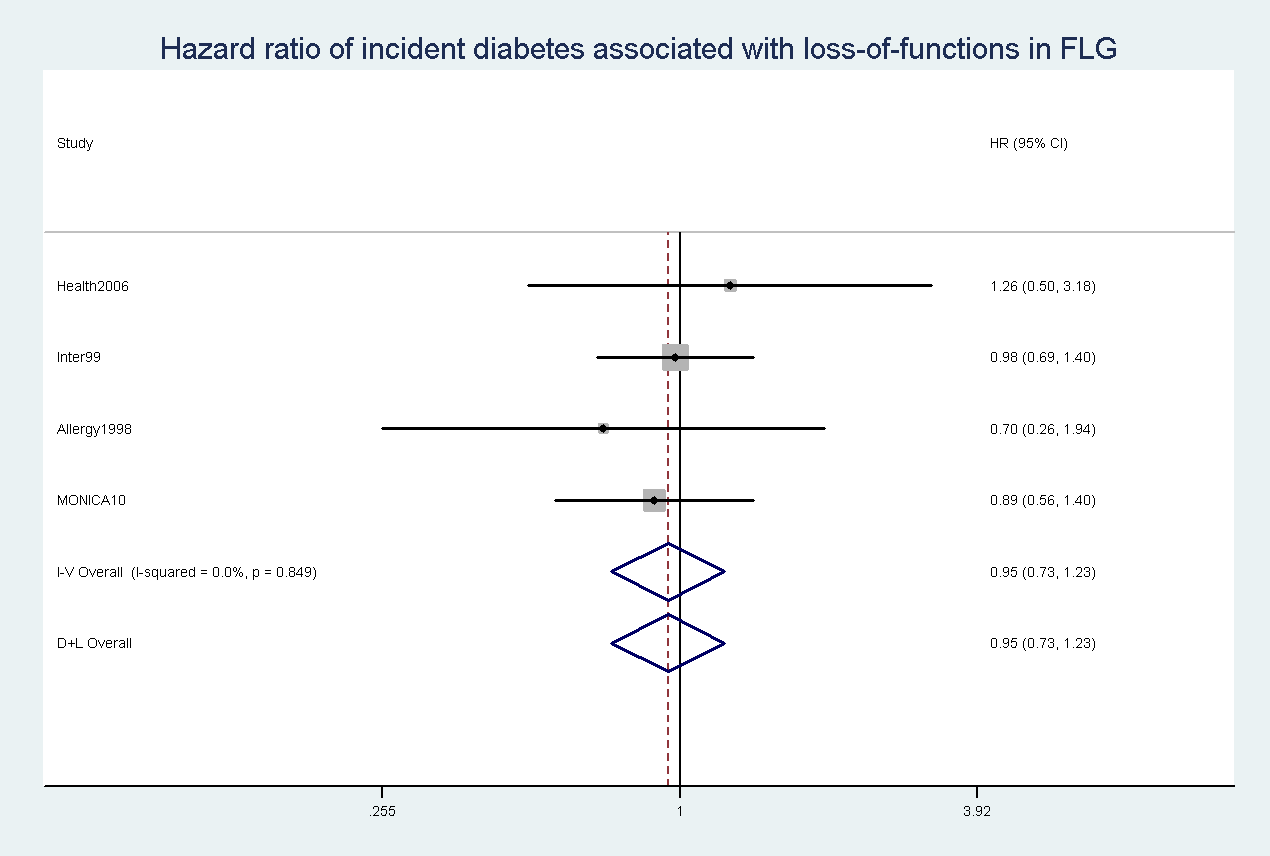

Supplement: Figure S1 — Hazard ratio of incident diabetes associated with loss-of-function mutations in FLG. (TIF) [file pone.0084293.s002.tif]

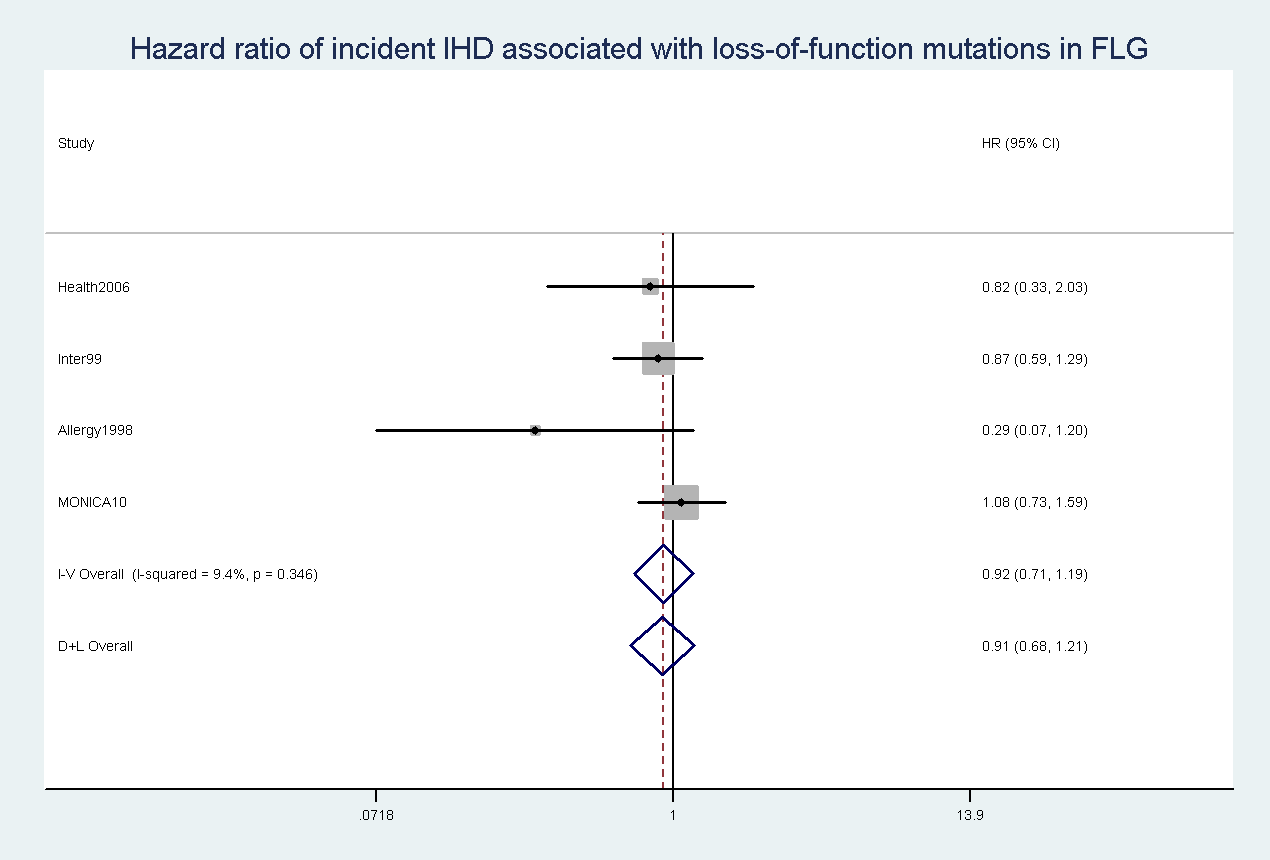

Supplement: Figure S2 — Hazard ratio of incident IHD associated with loss-of-function mutations in FLG. (TIF) [file pone.0084293.s003.tif]

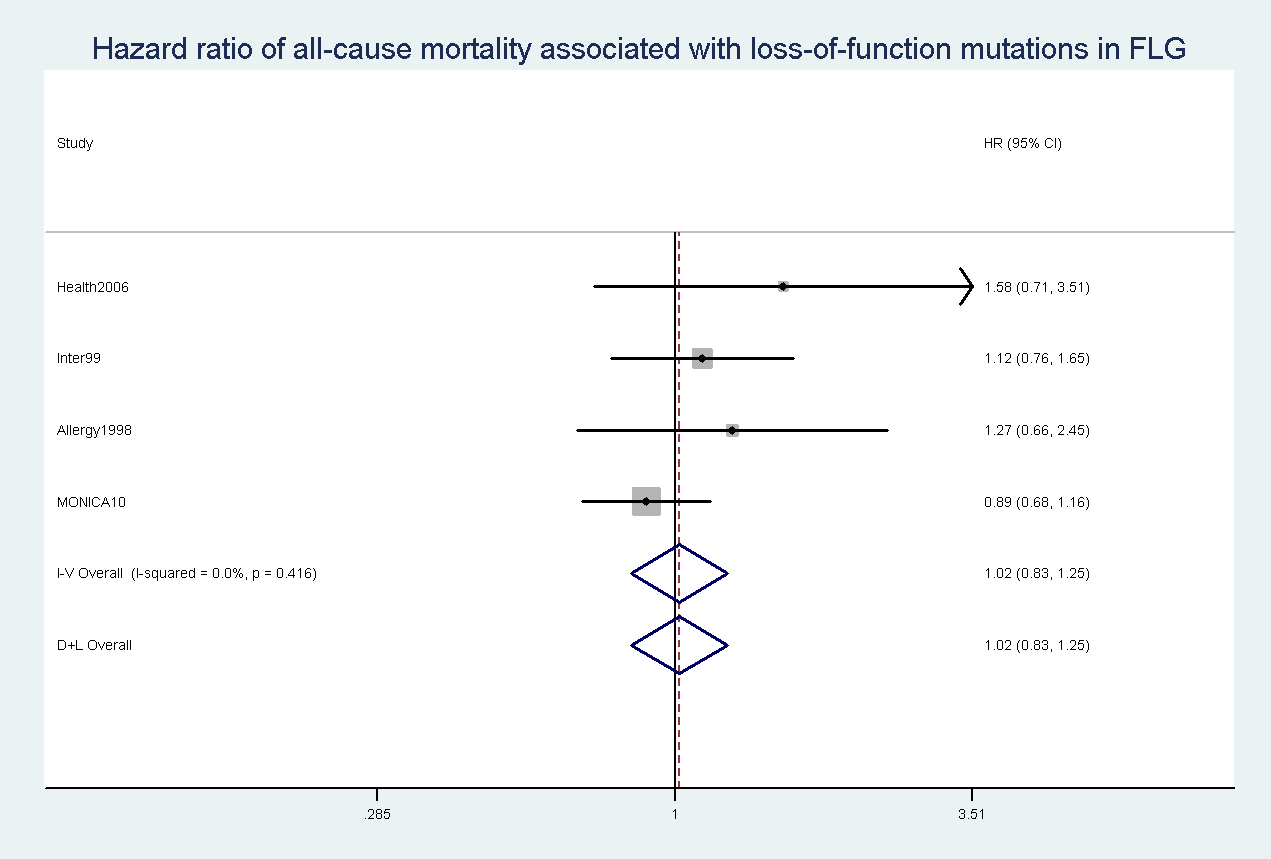

Supplement: Figure S3 — Hazard ratio of all-cause mortality associated with loss-of-function mutations in FLG. (TIF) [file pone.0084293.s004.tif]

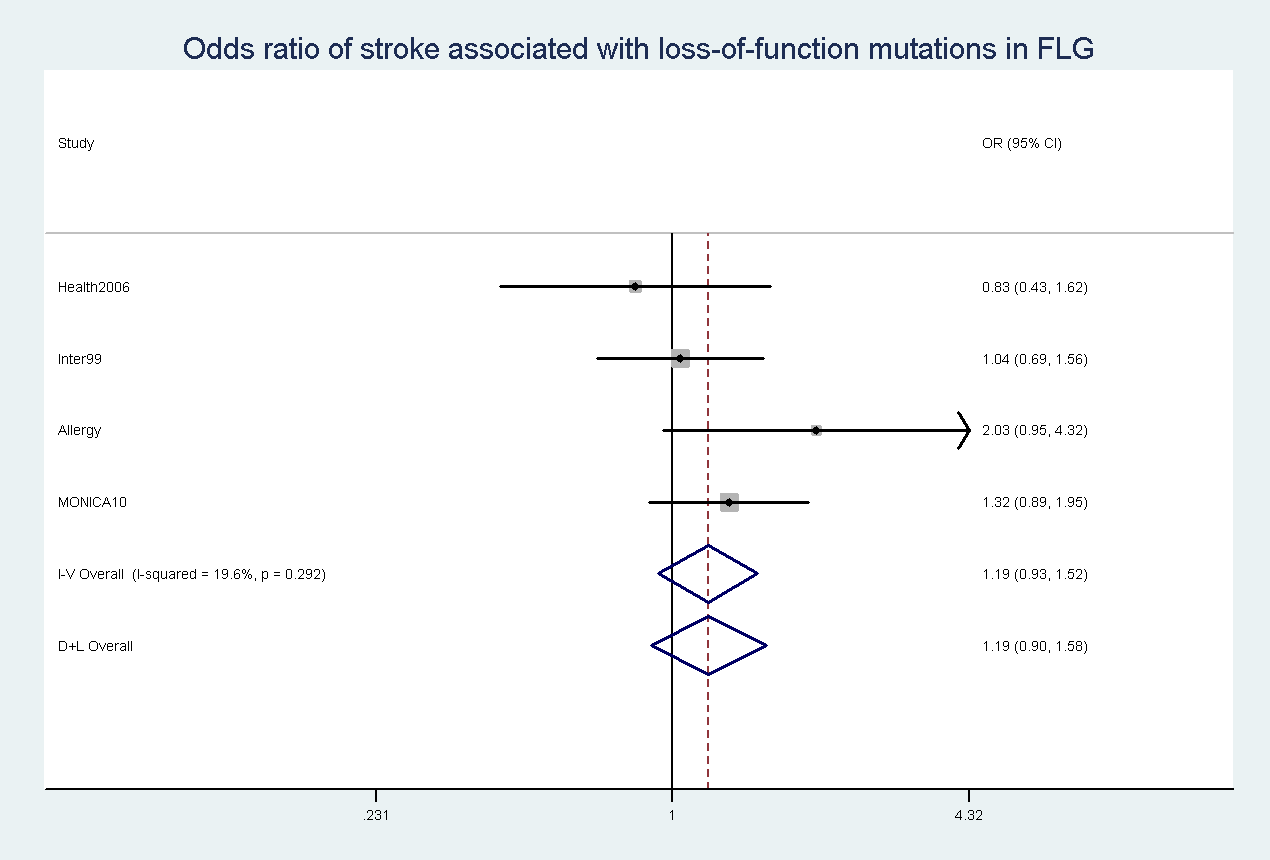

Supplement: Figure S4 — Odds ratio of stroke associated with loss-of-function mutations in FLG. (TIF) [file pone.0084293.s005.tif]

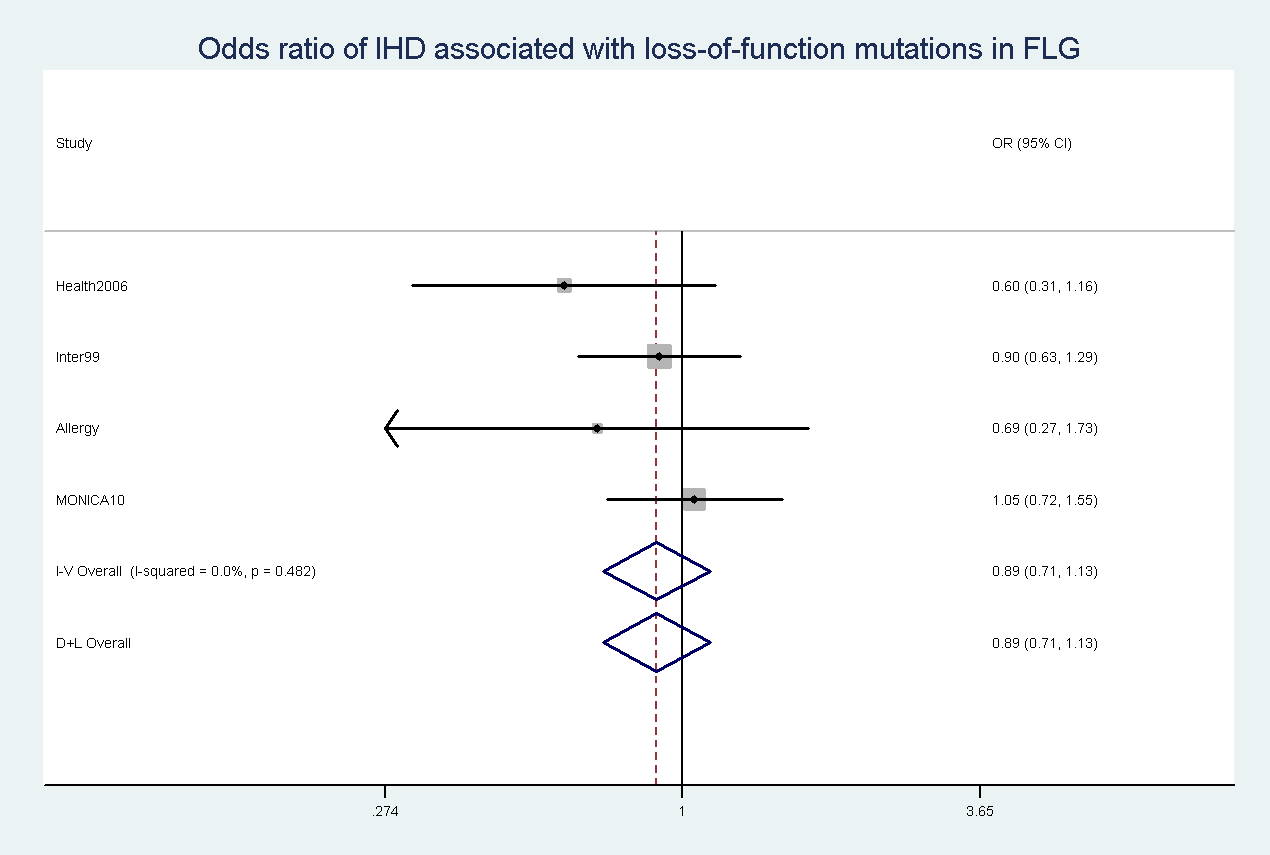

Supplement: Figure S5 — Odds ratio of IHD associated with loss-of-function mutations in FLG. (TIF) [file pone.0084293.s006.tif]
